# Beyond Colonization: Dynamic Risk Stratification and Clinical Decision-Making for MDR Gram-Negative Infections in Critically Ill Patients

**DOI:** 10.3390/biomedicines14081672

**Published:** 2026-07-24

**Authors:** Sara Palma Gullì, Rocco Morena, Francesca Serapide, Alessandro Russo

**Affiliations:** Infectious and Tropical Diseases Unit, Department of Medical and Surgical Sciences, “Magna Graecia” University of Catanzaro, 88100 Catanzaro, Italy; sarapalma08@gmail.com (S.P.G.); roccomorena1996@gmail.com (R.M.); f.serapide@unicz.it (F.S.)

**Keywords:** Gram-negative, colonization, infection, *Acinetobacter baumannii*, Enterobacterales, *Pseudomonas aeruginosa*, multidrug resistance

## Abstract

**Background**: Colonization with multidrug-resistant (MDR) Gram-negative bacteria is common in critically ill patients, yet the meaning of a positive surveillance culture is not always clear at the bedside. For some patients, colonization remains asymptomatic; for others, it may represent the first step toward an invasive infection that can develop rapidly. **Objectives**: This review examines the transition from colonization to infection in intensive care unit (ICU) patients carrying MDR *Pseudomonas aeruginosa*, carbapenem-resistant or extended-spectrum β-lactamase-producing Enterobacterales, and carbapenem-resistant *Acinetobacter baumannii* (CRAB). **Discussion**: Across these pathogens, colonization should not be viewed as an isolated microbiological finding. Its clinical relevance depends on the organism involved, site and persistence of carriage, extent of colonization, prior antibiotic exposure, severity of illness, invasive devices, impaired host defences, and the epidemiological pressure within the ICU. Intestinal carriage of resistant Enterobacterales is particularly relevant because it may act as an endogenous reservoir for subsequent invasive infection. In patients colonized with CRAB, respiratory and multisite carriage appear to identify those at greatest risk of pneumonia and poor outcomes. For *Pseudomonas aeruginosa*, the relationship is less uniform but becomes more relevant in the presence of respiratory vulnerability, ICU exposure, and previous broad-spectrum antibiotic use. **Conclusions**: Colonization results should therefore support risk stratification, infection-prevention measures, and informed empirical treatment when infection is suspected, rather than automatically leading to antibiotic therapy.

## 1. Introduction

Antimicrobial resistance represents a major global health threat [1]. In this review, MDR is defined according to the internationally accepted criteria proposed by Magiorakos et al., namely acquired non-susceptibility to at least one agent in three or more antimicrobial categories [2]. In critically ill patients, colonization by MDR pathogens poses a particular challenge because it may precede the development of difficult-to-treat infections. Colonization is generally defined as the asymptomatic carriage of bacteria resistant to commonly used antibiotics, detected through culture-based or molecular methods, and may represent a risk factor for subsequent infections [3].

However, the relationship between colonization and infection is not always linear or predictable. While this association is well established for some microorganisms, such as carbapenem-resistant Enterobacterales, it remains less clearly defined for others and may vary according to bacterial species, site of colonization, patient characteristics, and healthcare context. Gram-negative bacteria deserve particular attention due to their frequent antimicrobial resistance, especially to carbapenems, and their association with high rates of infection and mortality [2,4]. In the 2024 World Health Organization Bacterial Priority Pathogens List, carbapenem-resistant *Acinetobacter baumannii* and carbapenem-resistant or third-generation cephalosporin-resistant Enterobacterales were classified as critical-priority pathogens, whereas carbapenem-resistant *Pseudomonas aeruginosa* was included in the high-priority group [5].

Many studies conducted on different demographic and geographic cohorts have explored the association between colonization by MDR pathogens and adverse clinical outcomes, including infection development, increased mortality, and transmission [6,7]. However, these investigations are difficult to compare because they differ substantially in methodology, patient population, screening strategy, colonization site, microbiological definitions, and clinical endpoints [3,8,9,10,11,12,13]. Therefore, the magnitude of infection risk associated with previous colonization by MDR Gram-negative bacteria remains heterogeneous and incompletely defined across pathogens and clinical settings. Despite the growing number of publications, more robust clinical data are still needed to better define the adverse outcomes associated with colonization by resistant pathogens and to develop strategies to reduce infection-related mortality [14].

This review aims to examine the role of MDR Gram-negative colonization and the main factors that may facilitate progression to infection in critically ill patients. In particular, we focused on pathogens such as MDR *Pseudomonas aeruginosa*, carbapenem-resistant Enterobacterales, and *Acinetobacter baumannii*, analysing microbiological, patient-related, and environmental determinants.

## 2. Risk Factors

The progression from colonization to infection in critically ill patients colonized by MDR Gram-negative bacteria is a multifactorial process. Rather than being determined by colonization status alone, the risk of infection depends on the interaction between pathogen-related determinants, host susceptibility, and healthcare-associated environmental pressures. These factors may act simultaneously and amplify one another, particularly in ICU settings, where patients are frequently exposed to invasive devices, broad-spectrum antibiotics, prolonged hospitalization, and high colonization pressure [3,13,14,15].

For this reason, colonization should be interpreted within a dynamic risk-stratification framework. The same microbiological finding may have different clinical implications depending on the bacterial species involved, the anatomical site and burden of colonization, the presence of multisite colonization, the severity of the underlying disease, the integrity of host defenses, and the surrounding epidemiological context. A rectal swab positive for carbapenem-resistant Enterobacterales, respiratory colonization by carbapenem-resistant *Acinetobacter baumannii*, or gastrointestinal carriage of MDR *Pseudomonas aeruginosa* may therefore confer different levels of infection risk according to the clinical setting [3,13,14]. Table 1 summarizes the clinical interpretation and management implications of MDR Gram-negative colonization in critically ill patients.

**Figure 1:**
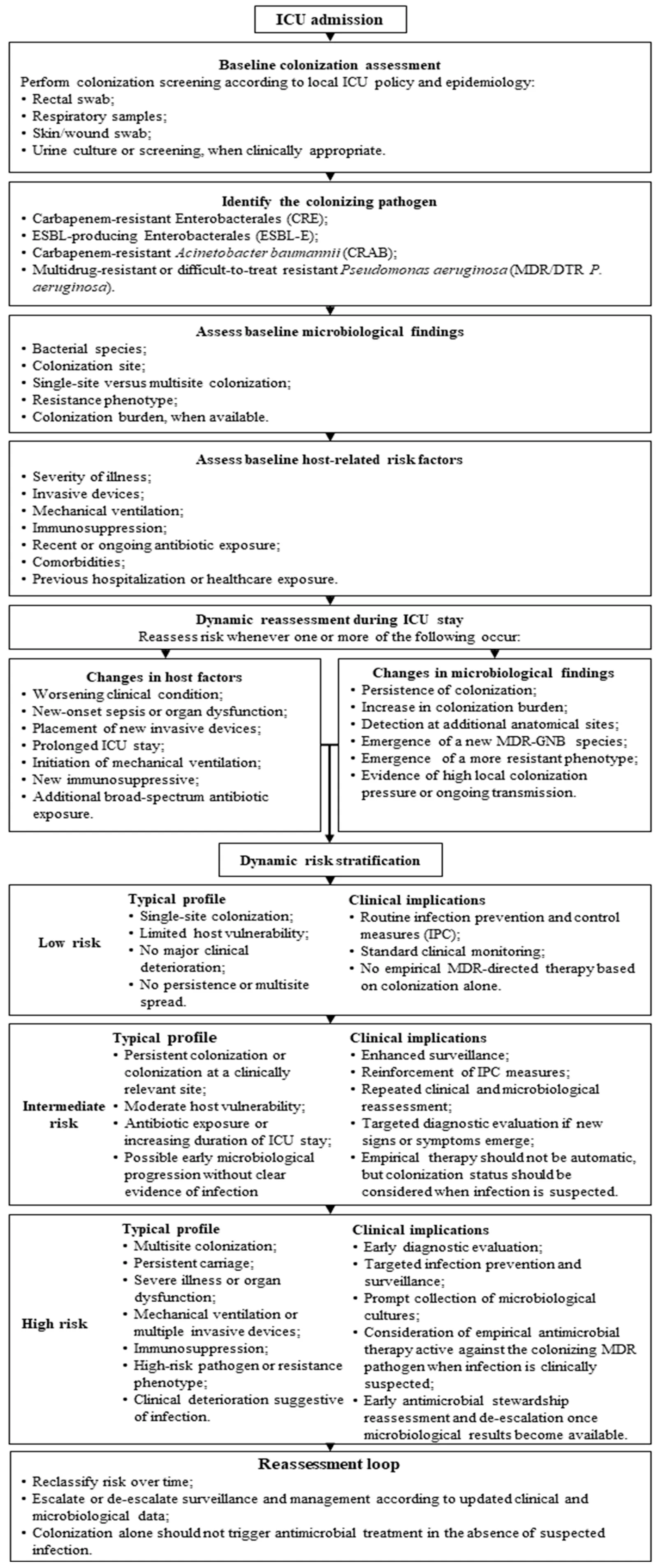
Dynamic risk stratification framework for critically ill patients colonized with MDR Gram-negative bacteria.

Dynamic risk stratification should be viewed as a repeated bedside assessment rather than as the interpretation of a single surveillance result [3,13,14]. The colonizing pathogen, colonization site, and baseline host vulnerability should be reassessed during the ICU stay according to clinical deterioration, invasive devices, antibiotic exposure, persistence, or multisite colonization. This conceptual low-, intermediate-, and high-risk framework may guide surveillance, infection prevention and control measures, diagnostic evaluation, and empirical MDR-active therapy when infection is clinically suspected. Colonization alone should not prompt antimicrobial treatment (Figure 1).

## 3. Microbiological Factors

Microbiological determinants play a central role in modulating the risk of progression from colonization to infection [34]. Beyond the mere presence of MDR Gram-negative bacteria, the probability of developing clinically significant disease may vary according to bacterial species, resistance phenotype, site of colonization, bacterial burden, persistence over time, biofilm-forming capacity, environmental survival, virulence traits, and the ability to colonize multiple anatomical sites. Representative examples include the type III secretion system, ExoU and ExoS effectors, and quorum-sensing pathways in *Pseudomonas aeruginosa*, as well as biofilm-associated proteins and adhesion mechanisms in *Acinetobacter baumannii*. These characteristics are particularly relevant in critically ill patients, in whom impaired host defenses and intensive healthcare exposure may facilitate the transition from asymptomatic carriage to invasive infection [35,36,37].

Among MDR Gram-negative bacteria, *Pseudomonas aeruginosa*, Enterobacterales, and *Acinetobacter baumannii* differ substantially in their epidemiology, preferred colonization niches, mechanisms of persistence, and strength of association between colonization and subsequent infection. Therefore, the microbiological contribution to infection risk should be analyzed separately for each pathogen group. Accordingly, the following sections distinguish studies evaluating the acquisition of colonization, progression from colonization to infection, and outcomes among patients with established infection, as these address related but clinically distinct questions.

### 3.1. Pseudomonas aeruginosa

*Pseudomonas aeruginosa* is a Gram-negative pathogen frequently responsible for healthcare-associated infections with mortality rates ranging from 10.5% to 71%. This broad range likely reflects differences in infection site, patient population and comorbidity burden, baseline severity of illness, antimicrobial resistance phenotype, appropriateness of antimicrobial therapy, and study design [38,39,40]. Its intrinsic resistance mechanisms, ability to acquire additional resistance determinants, biofilm-forming capacity, and adaptation to hospital environments contribute to its persistence in high-acuity settings and limit therapeutic options [41].

Throughout this section, multidrug-resistant (MDR) *Pseudomonas aeruginosa* is defined according to the Magiorakos criteria [2]. Difficult-to-treat resistant (DTR) *Pseudomonas aeruginosa* refers to isolates showing non-susceptibility to standard first-line antipseudomonal agents, particularly carbapenems, extended-spectrum cephalosporins, and fluoroquinolones [42].

Several studies have identified risk factors for *Pseudomonas aeruginosa* infection, including prior exposure to broad-spectrum antibiotics, recent hospitalization or ICU stay, prolonged length of stay, mechanical ventilation, vasopressor support, and previous colonization or infection [22,43]. Additionally, patient-specific conditions, such as uncontrolled type II diabetes mellitus, severe COPD, bronchiectasis, cystic fibrosis, bedridden status, and renal failure, have been associated with an increased risk of acquiring MDR *Pseudomonas aeruginosa* infections [42,43]. Despite this knowledge, evidence defining the specific contribution of colonization to subsequent infection remains limited and heterogeneous. These gaps underscore the need for a clearer understanding of how colonization influences the progression to infection, especially in critically ill patients. In the ICU setting, where early appropriate antimicrobial therapy is crucial, knowledge of colonization status may inform clinical decision-making [23].

A study conducted in Maryland evaluated the impact of *Pseudomonas aeruginosa* colonization in patients admitted to the ICU, assessing its prevalence, associated risk factors, and clinical outcomes. Among the 1840 patients screened at ICU admission, 213 patients (11.6%) were colonized, indicating that approximately one in nine patients already carried this organism at the time of hospitalization. Risk factors associated with colonization included advanced age, anemia, and underlying neurological conditions. Among colonized patients, 28.2% subsequently developed a clinical culture positive for *Pseudomonas aeruginosa* during hospitalization, either in the ICU or in the following inpatient period. Most of these cases represented clinically significant infections, mainly pneumonia, urinary tract infections, wound infections, bloodstream infections (BSI), and intra-abdominal infections. In contrast, only 4.2% of non-colonized patients developed a positive culture during hospitalization. These proportions correspond to a calculated unadjusted risk ratio of 6.74, indicating an approximately 6.7-fold higher risk of a subsequent positive clinical culture among colonized patients. Of the 60 colonized patients with a subsequent positive culture, 49 (81.7%) were considered to have a clinically significant infection, including pneumonia, urinary tract infection, wound infection, BSI, or intra-abdominal infection. These findings suggest that colonization at ICU admission may identify a subgroup of patients at substantially higher risk of subsequent infection [23].

The intestinal tract may also represent an important reservoir for *Pseudomonas aeruginosa* in critically ill patients. In a comparative study of fecal samples from ICU inpatients and healthy individuals, intestinal colonization was more frequent among ICU patients, and isolates from critically ill patients showed higher rates of antimicrobial resistance, including carbapenem resistance [25,26]. Interestingly, the population structure was polyclonal, suggesting multiple independent acquisition events rather than expansion of a single clone. Moreover, ICU isolates showed a stronger resistance profile, whereas virulence gene distribution did not necessarily parallel resistance, highlighting the complex relationship between antimicrobial resistance, fitness, and pathogenic potential [25,27].

Clinical studies focusing on DTR *Pseudomonas aeruginosa* infections further support the relevance of healthcare exposure and respiratory involvement in risk assessment. In a multicenter study conducted in Lebanese tertiary hospitals, DTR *Pseudomonas aeruginosa* infections were independently associated with ICU stay and a respiratory source of infection [22]. These findings suggest that ICU exposure and respiratory involvement may help identify patients at increased risk of infection due to DTR *Pseudomonas aeruginosa*.

Overall, *Pseudomonas aeruginosa* colonization should not be interpreted as a uniform predictor of infection. Respiratory colonization, persistence of carriage, respiratory vulnerability, and healthcare exposure appear most relevant to progression risk, whereas virulence determinants mainly influence pathogenicity and disease severity after infection has occurred [23,24,35]. Colonization status may therefore inform respiratory surveillance and empirical antipseudomonal therapy when infection is clinically suspected, followed by prompt microbiological reassessment and de-escalation.

### 3.2. Enterobacterales

#### 3.2.1. Epidemiology of CRE and MDR Enterobacterales

Multidrug-resistant bacteria that pose a major public health threat and require targeted prevention programs also include Enterobacterales, particularly carbapenem-resistant phenotypes (CRE). Among these, carbapenem-resistant *Klebsiella pneumoniae* appears to be the most frequent cause of serious infections and is responsible for hospital outbreaks. However, carbapenem resistance has also been reported in *Klebsiella oxytoca*, *Escherichia coli*, and *Enterobacter cloacae* [44,45].

In recent decades, the incidence rates of MDR Enterobacterales have progressively increased globally. Italy represents a particularly relevant setting, being considered a highly endemic area for CRE, largely driven by KPC-producing *Klebsiella pneumoniae*. National surveillance data have reported high rates of BSI caused by carbapenem-resistant *Klebsiella pneumoniae*, reaching 26.7% in 2021 [46]. Regarding CRE colonization rates, it is believed that in endemic areas, prevalence in hospitalized patients ranges from 3 to 7%, with even higher rates reported among patients admitted to the ICU [47].

#### 3.2.2. Intestinal Colonization and Progression to Infection

The gastrointestinal tract is the main reservoir of Enterobacterales and plays a central role in the progression from colonization to invasive infection. Although Enterobacterales may also colonize the oropharynx, skin, and urinary tract, intestinal carriage is particularly relevant because it may persist for prolonged periods and provide an endogenous source for subsequent invasive disease [48,49,50,51]. Although the likelihood of infection among CRE-colonized individuals is variable, early identification of colonization status is considered essential to limit CRE transmission and implement timely infection prevention and control measures [50,51]. Therefore, colonization data may support clinical decision-making by guiding isolation or cohorting strategies, reinforcing surveillance in high-risk units, and informing empirical antimicrobial choices in selected critically ill patients [16,51].

Other recently published studies have analysed the role of Enterobacterales intestinal colonization as a prognostic factor for invasive infections in hospitalized patients admitted to the ICU. In a large retrospective cohort including 7006 hospitalized patients screened by rectal swab, 11.9% were colonized with MDR Enterobacterales at admission, and 6.1% subsequently developed nosocomial BSI caused by Enterobacterales. Colonized patients had a significantly higher risk of BSI than non-colonized patients, and this association was observed both for ESBL-producing Enterobacterales and for carbapenem-resistant strains [17]. These findings support the concept that the gut may act as an endogenous reservoir from which MDR Enterobacterales can translocate and cause invasive infection, particularly when host defenses and mucosal barriers are impaired.

#### 3.2.3. Impact of COVID-19 on CRE Epidemiology

The importance of intestinal colonization is further supported by epidemiological data from highly endemic settings. A multicenter study conducted in Italy between 2018 and 2020 examined the trend in intestinal colonization by CRE before and during the first year of the COVID-19 pandemic. The survey involved over 11,000 hospitalized patients in seven different hospitals and used a systematic screening protocol based on rectal swabs performed at the time of admission and repeated in the following days for patients who initially tested negative. The analysis showed that, even in the pre-pandemic period, the prevalence of CRE colonization at admission ranged from 3.9% to 11.5%, while hospital-acquired colonization ranged from approximately 5% to 15%. During the first year of the pandemic, these values increased, showing a significant rise in both patients already colonized at admission and new cases acquired during hospitalization. This increase was not uniform across the country: hospitals in southern Italy showed the most pronounced increase, with some departments, particularly ICUs, reporting very high percentages of CRE-positive patients at admission. In contrast, some hospitals in northern Italy showed no significant changes and, in some cases, even a reduction in hospital-acquired colonization. From a microbiological perspective, most isolates were multidrug-resistant *Klebsiella pneumoniae*. Overall, the study suggests that the first year of the COVID-19 pandemic contributed to increased CRE colonization, likely due to organizational pressures, increased healthcare workload, and difficulties maintaining infection prevention and control practices. These findings underscore the need for surveillance and prevention strategies tailored to local epidemiological contexts, given the considerable heterogeneity observed [52].

#### 3.2.4. Colonization Resistance and the Gut Microbiome

Another study addresses a central concept, namely “resistance to colonization” [53]. The review emphasizes that colonization is a multifactorial phenomenon resulting from the interaction between microbial, host, and environmental determinants. Microbial factors include the presence of resistance plasmids, adhesion structures, and horizontal gene transfer capabilities. Host factors include mucosal barrier integrity, antimicrobial peptide production, and local immune responses. Environmental and clinical pressures, particularly exposure to antibiotics, hospitalization, procedural interventions, and physiological stress, further modulate the microbiome [20,53]. Overall, the evidence highlights that intestinal colonization by MDR Enterobacterales is a dynamic and complex process, strongly influenced by the composition and functional capacity of the gut microbiota. The presence of specific commensals, particularly within Lachnospiraceae, appears to confer protection against MDR colonization, highlighting the potential of microbiome-targeted strategies, including judicious use of antibiotics, probiotics, fecal microbiota transplantation, and dietary modulation, to prevent or mitigate colonization. However, further studies are needed to clarify the causal relationships between microbiome disturbances and MDR colonization and to evaluate microbiota-based interventions [53].

In a cohort of 255 critically ill patients evaluated before antibiotic exposure, fecal carriage of ESBL-producing Enterobacterales was mainly due to ESBL-producing *Escherichia coli* and *Klebsiella pneumoniae*. Importantly, ESBL-producing *Klebsiella pneumoniae* carriers showed reduced intestinal bacterial diversity compared with ESBL-producing *Escherichia coli* carriers and non-carriers. Specific bacterial and fungal taxa were also associated with either the presence or absence of ESBL-producing Enterobacterales, suggesting that colonization risk may depend not only on the resistance phenotype, but also on the bacterial species and the surrounding microbial ecosystem. For example, the presence of Sellimonas intestinalis was associated with the absence of ESBL *Escherichia coli*, while the presence of *Campylobacter ureolyticus*, *Campylobacter hominis* and some *Saccharomyces species* was associated with the absence of ESBL *Klebsiella pneumoniae*. Conversely, a higher abundance of *Candida albicans* was associated with the presence of ESBL *Escherichia coli* in some patients [54].

#### 3.2.5. Clinical Implications and Future Perspectives

Taken together, these findings indicate that intestinal colonization by CRE or ESBL-producing Enterobacterales represents one of the clearest examples of how asymptomatic carriage can translate into clinically relevant infection risk [18,21]. Compared with *Pseudomonas aeruginosa*, for which the colonization-infection relationship appears more heterogeneous, the association between intestinal carriage of MDR Enterobacterales and subsequent invasive infection is more consistently supported by available evidence. Nevertheless, the magnitude of this risk remains dynamic and depends on the interaction between resistance phenotype, bacterial species, colonization burden, gut microbiota composition, patient severity, antibiotic exposure, and local epidemiology.

Intestinal carriage, persistence of colonization, resistance phenotype, impaired mucosal barriers, and severe underlying illness are the main factors informing the risk of progression to invasive infection. Identifying MDR Enterobacterales colonization may therefore support early risk stratification, targeted infection prevention measures, and empirical antimicrobial choices in selected high-risk patients, but should not prompt antimicrobial treatment in the absence of clinical infection [17,18,21,55]. The role of dysbiosis and impaired colonization resistance also reinforces the importance of antimicrobial stewardship and raises interest in microbiota-modulating strategies aimed at reducing colonization burden [56].

### 3.3. Acinetobacter baumannii

Among non-fermenting Gram-negative bacteria, multidrug-resistant *Acinetobacter baumannii* represents one of the most challenging healthcare-associated pathogens and is responsible for outbreaks and severe infections among critically ill patients [57]. Carbapenem-resistant *Acinetobacter baumannii* (CRAB) is of particular concern because of its extensive antimicrobial resistance, environmental persistence, and limited therapeutic options. Distinguishing colonization from infection may be difficult, particularly when the organism is isolated from respiratory specimens, and therefore requires careful integration of microbiological and clinical findings [28,29].

Available evidence indicates that CRAB colonization should not be regarded as a benign or incidental microbiological finding, although its predictive value is not uniform. Surveillance cultures positive for CRAB have been associated with an increased risk of subsequent clinically significant infection [58]. Progression is influenced by the anatomical site, persistence and burden of colonization, the presence of multisite colonization, mechanical ventilation, invasive devices, previous exposure to broad-spectrum antibiotics, prolonged ICU stay, immunosuppression, underlying comorbidities, and colonization pressure within the unit [28,29,31,58,59]. The ability of *Acinetobacter baumannii* to form biofilms, persist on environmental surfaces, and express virulence and immune-evasion factors further facilitate prolonged carriage and cross-transmission [31,59].

The importance of integrated preventive strategies is further supported by a study conducted during a CRAB outbreak in an ICU. A five-component infection control bundle, including reinforcement of hand hygiene, expanded microbiological screening, universal contact precautions, enhanced environmental sampling, and a radical environmental cleaning and disinfection procedure, reduced the incidence density of nosocomial CRAB colonization or infection from 30.4 to zero cases per 1000 patient-days [32]. Whole-genome sequencing also demonstrated a clonal relationship between clinical and environmental isolates, highlighting the central role of environmental reservoirs in CRAB transmission. These findings support the implementation of multimodal infection prevention and control strategies to limit CRAB acquisition and spread in intensive care settings [32].

The importance of the colonization site and burden was further demonstrated in a multicenter retrospective study of 760 critically ill patients with respiratory CRAB colonization [28]. Hospital-acquired pneumonia (HAP) developed in 51.5% of patients, while 39.9% had CRAB detected at more than one anatomical site. Multisite colonization was independently associated with progression to HAP and was more common among patients who subsequently developed HAP. Other predictors included advanced age, immunosuppressive treatment, chronic obstructive pulmonary disease, and the need for ventilatory support. In-hospital mortality reached 76.3%, and multisite colonization was associated with reduced short-term and overall survival [28]. These findings suggest that respiratory colonization accompanied by multisite carriage identifies a particularly vulnerable subgroup, rather than establishing that every CRAB-positive respiratory culture represents infection.

Although *Acinetobacter baumannii* is predominantly associated with healthcare environments, recent studies have also identified MDR isolates among companion animals with respiratory disease and carbapenem-resistance genes among isolates obtained from broiler chickens [60,61]. These observations support a One Health perspective and suggest the possible existence of extra-hospital reservoirs. Nevertheless, their direct contribution to CRAB acquisition and infection among hospitalized patients remains uncertain and requires further genomic and epidemiological investigation.

These findings support a proactive approach to CRAB management, moving beyond the treatment of established infections. Effective strategies should integrate active surveillance, early risk stratification, rigorous infection prevention and control measures, and antimicrobial stewardship [62]. Although interventions such as chlorhexidine bathing may be promising, evidence on decolonization and the cost-effectiveness of surveillance programs remains limited [63]. Further studies are needed to refine predictive tools and optimize integrated strategies aimed at reducing colonization, limiting transmission, and improving outcomes.

Respiratory and multisite colonization, persistence of carriage, mechanical ventilation, and colonization pressure are particularly relevant to progression risk, whereas biofilm formation and environmental persistence mainly contribute to prolonged carriage and cross-transmission. These findings support intensified surveillance, rigorous infection prevention measures, and early diagnostic evaluation during clinical deterioration; empirical CRAB-active therapy should remain restricted to patients with suspected infection [28,29,31,32,58,59,62,63].

## 4. Risk Stratification Models and Predictive Factors: The Giannella Score and the Cogliati-Dezza Study

In recent years, increasing attention has been devoted to predictive tools aimed at identifying colonized patients at higher risk of progression to infection. In the setting of carbapenem-resistant Gram-negative bacteria, such models may help move clinical management beyond the simple detection of colonization toward a more structured assessment of individual risk. Among the available approaches, the Giannella Risk Score represents a formal prediction tool for carbapenem-resistant *Klebsiella pneumoniae*, whereas the study by Cogliati-Dezza et al. identified CRAB-specific predictors without deriving or validating a formal clinical score.

The Giannella Risk Score (GRS) was originally developed to predict the risk of BSI in patients colonized with carbapenem-resistant *Klebsiella pneumoniae*. It includes clinical variables independently associated with progression from colonization to invasive infection, including multisite colonization, recent endoscopic procedures, central venous catheterization, ICU stay, and recent antibiotic exposure. Higher GRS values correlate with a significantly increased likelihood of subsequent carbapenem-resistant *Klebsiella pneumoniae* BSI [19]. Although this score was not specifically designed for *Acinetobacter baumannii*, the conceptual relevance extends beyond MDR *Klebsiella pneumoniae.* Indeed, several of its core variables, like colonization burden, invasive procedures, and clinical severity, are also consistently recognized as determinants of infection risk in patients colonized with CRAB. For this reason, the GRS may be considered a useful methodological reference when discussing risk stratification in CRAB-colonized patients, rather than a directly validated tool for this specific pathogen [19,64].

More recently, Cogliati-Dezza et al. prospectively evaluated 129 ICU patients colonized with CRAB, 57 of whom developed CRAB bloodstream infection. Higher Charlson Comorbidity Index, COVID-19, multisite colonization, and mechanical ventilation were independently associated with BSI development [33]. Unlike the GRS, this study did not derive or validate a formal clinical score; rather, it identified pathogen-specific predictors that may support bedside risk assessment in CRAB-colonized patients.

Despite their differences, both approaches emphasize multisite colonization and clinical vulnerability as important determinants of progression from colonization to infection. These shared elements are consistent with the broader literature on CRAB, in which colonization is increasingly interpreted not as a passive microbiological state, but as a dynamic risk condition influenced by host vulnerability, healthcare exposure, and pathogen persistence [19,33].

The integration of validated scores and pathogen-specific predictive factors into clinical practice may offer two main advantages. First, it could support risk-guided surveillance, allowing intensified microbiological monitoring and infection preventive measures in patients with higher predicted risk. Second, it may help clinicians anticipate clinical deterioration and guide empirical therapy decisions when infection is suspected, while still preserving antimicrobial stewardship principles. Although the GRS has not been universally adopted, CRAB-specific predictive factors still require external validation. Future studies are therefore needed to refine these models, assess their performance in different ICU populations, and develop predictive tools specifically tailored to CRAB colonization and infection progression.

## 5. Conclusions

The available evidence suggests that colonization by MDR Gram-negative bacteria may serve as a clinically relevant marker of subsequent infection risk in critically ill patients, although its predictive value varies considerably according to the pathogen, colonization site, patient characteristics, and clinical setting. Colonization may precede the development of severe infection, but it should not be regarded as an inevitable step toward invasive disease. The association appears more consistently supported for carbapenem-resistant or ESBL-producing Enterobacterales and CRAB, whereas the available evidence is more heterogeneous for MDR *Pseudomonas aeruginosa* [3,14,18,24,28,64].

In intensive care settings, colonization is highly prevalent, often multisite, and frequently involves highly resistant strains. This carrier state may act as an endogenous reservoir, from which pathogens may translocate when host defences are compromised by intestinal dysbiosis, mucosal barrier impairment, or invasive devices. Several studies show that the transition from colonization to infection can occur within a short time frame, particularly in clinically unstable patients, resulting in significantly higher mortality, prolonged hospitalization, and increased healthcare resource utilization.

Colonization may also play a crucial role in intra-hospital transmission. High colonization pressure, environmental contamination, and cross-transmission through healthcare workers can sustain the circulation of MDR organisms within ICUs and other high-risk wards. Thus, colonization could represent not only an individual clinical risk but also a key epidemiological driver of MDR spread within ICUs and hospital wards.

An additional and increasingly recognized dimension is the relationship between colonization and the gut microbiota. Reduced microbial diversity and the loss of specific commensal taxa predispose patients to colonization by MDR pathogens, while a richer and more stable microbiota appears to confer colonization resistance. This supports a possible role of microbiota disruption, common in critically ill patients and frequently exacerbated by antibiotic exposure, in facilitating colonization and potentially increasing the risk of infection.

Taken together, these findings support a shift from a purely reactive approach, focused only on treating established infections, to a proactive strategy aimed at preventing, detecting, and controlling colonization. Active surveillance programs, rigorous infection prevention and control measures, antimicrobial stewardship, and emerging microbiota-modulating interventions are essential to identify colonized patients early and prevent progression to invasive infection. Structured prediction tools and pathogen-specific predictors, such as the Giannella Score and those identified by Cogliati-Dezza et al., further illustrate the potential value of risk stratification in guiding targeted preventive measures.

In conclusion, colonization may represent a clinically relevant and context-dependent marker of infection risk in critically ill patients carrying MDR Gram-negative bacteria. Early recognition and targeted intervention are essential to reduce infection risk, limit transmission, and improve outcomes in high-acuity healthcare settings. The main studies on progression from colonization to infection are summarized in Table 2.

## Figures and Tables

**Table 1:** Clinical interpretation and management implications of MDR Gram-negative colonization in critically ill patients.

Colonization Finding	Risk Interpretation	Factors Increasing Concern	Possible Clinical Action	Key References
Rectal CRE colonization	High relevance, especially for subsequent BSI	CVC, ICU stay, recent antibiotics, endoscopy, immunosuppression, high local endemicity	Contact precautions, targeted surveillance, consider CRE coverage if sepsis develops	[16,17,18,19]
ESBL Enterobacterales carriage	Moderate-to-high relevance depending on setting	Prior antibiotics, dysbiosis, ICU admission, severe illness	Stewardship, avoid unnecessary carbapenems, consider risk in empirical therapy	[20,21]
Respiratory MDR *Pseudomonas aeruginosa* colonization	Context-dependent but clinically relevant	Mechanical ventilation, COPD/bronchiectasis, previous antibiotics, respiratory deterioration	Consider in VAP/HAP empirical therapy; reassess with cultures	[22,23,24]
Gastrointestinal MDR *Pseudomonas aeruginosa* carriage	Potential reservoir, less clearly predictive	ICU stay, antibiotic pressure, high resistance phenotype	Surveillance in high-risk settings; avoid overinterpretation without clinical signs	[25,26,27]
Respiratory CRAB colonization	High concern in ICU	Ventilation, COPD, immunosuppression, multisite colonization, colonization pressure	Strict IPC, close monitoring, early diagnostic work-up if deterioration occurs	[28,29,30]
Multisite CRAB colonization	Very high-risk marker	Multiple positive sites, ventilatory support, severe illness	Intensified surveillance, isolation/cohorting, environmental control, stewardship	[28,31,32,33]

Legend: BSI: bloodstream infection; COPD: chronic obstructive pulmonary disease; CRAB: carbapenem-resistant *Acinetobacter baumannii*; CRE: carbapenem-resistant Enterobacterales; CVC: central venous catheter; ESBL: extended-spectrum β-lactamase; HAP: hospital-acquired pneumonia; ICU: intensive care unit; IPC: infection prevention and control; MDR: multidrug-resistant; VAP: ventilator-associated pneumonia.

**Table 2:** Key studies evaluating progression from colonization to infection among MDR Gram-negative carriers.

Study	Pathogen	Population	Colonization Site	Main Findings	Clinical Relevance	Key References
Harris et al.	*Pseudomonas aeruginosa*	ICU patients	Screening at ICU admission	11.6% colonized; 28.2% developed positive clinical cultures vs. 4.2% non-colonized	Colonization identifies patients at higher risk, but association remains context-dependent	[23]
Hu et al.	*Pseudomonas aeruginosa*	ICU patients vs. healthy individuals	Gut/fecal samples	Higher colonization and resistance among ICU patients	Gut may act as reservoir for MDR *Pseudomonas aeruginosa*	[25]
Henoun Loukili et al.	MDR Enterobacterales	Hospitalized patients	Rectal swab	Colonized patients had higher risk of nosocomial Enterobacterales BSI	Supports gut reservoir → BSI model	[17]
Russo et al.	CRAB	Critically ill patients	Respiratory ± multisite	51.5% developed HAP; multisite colonization independently predicted HAP	Multisite colonization is a key ICU risk marker	[28]
Giannella et al.	CR-*Klebsiella pneumoniae*	Rectal carriers	Rectal/multisite	Identified predictors of BSI including multisite colonization, CVC, ICU stay, antibiotics	Basis for structured risk prediction	[19]
Peghin et al.	CRAB	Colonized patients	Various sites	27.8% progressed to infection; median 4 days	CRAB colonization may rapidly evolve in high-risk patients	[65]

Legend: BSI: bloodstream infection; CRAB: carbapenem-resistant *Acinetobacter baumannii*; CR-*Klebsiella pneumoniae*: carbapenem-resistant *Klebsiella pneumoniae*; HAP: hospital-acquired pneumonia; ICU: intensive care unit; MDR: multidrug-resistant.

## Data Availability

No new data were created or analyzed in this study. Data sharing is not applicable to this article.
